# Practical recommendations for biochemical and genetic diagnosis of the porphyrias

**DOI:** 10.1111/liv.16012

**Published:** 2024-06-28

**Authors:** Aasne K. Aarsand, Jordi To‐Figueras, Sharon Whatley, Sverre Sandberg, Caroline Schmitt

**Affiliations:** ^1^ Norwegian Porphyria Centre and Department of Medical Biochemistry and Pharmacology Haukeland University Hospital Bergen Norway; ^2^ Norwegian Organization for Quality Improvement of Laboratory Examinations (NOKLUS) Haraldsplass Deaconess Hospital Bergen Norway; ^3^ Biochemistry and Molecular Genetics Unit Hospital Clinic‐University of Barcelona Barcelona Spain; ^4^ Cardiff Porphyria Service, Department of Medical Biochemistry and Immunology University Hospital of Wales Healthcare NHS Trust Cardiff UK; ^5^ Department of Global Public Health and Primary Care, Faculty of Medicine University of Bergen Bergen Norway; ^6^ Department of Medical Biochemistry Université Paris Cité and INSERM U1149, Centre de Recherche sur l'Inflammation Paris France; ^7^ French Centre of Porphyrias, Assistance Publique‐Hôpitaux de Paris Hôpital Louis Mourier Colombes France

**Keywords:** biomarkers, genetic testing, haeme, inborn error metabolism, porphyria, porphyrins

## Abstract

The porphyrias are a group of rare inborn errors of metabolism associated with various clinical presentations and long‐term complications, making them relevant differential diagnoses to consider for many clinical specialities, especially hepatologists, gastroenterologists and dermatologists. To diagnose a patient with porphyria requires appropriate biochemical investigations, as clinical features alone are not specific enough. Furthermore, it is important to be aware that abnormalities of porphyrin accumulation and excretion occur in many other disorders that are collectively far more common than the porphyrias. In this review, we provide an overview of porphyria‐related tests with their strengths and limitations, give recommendations on requesting and diagnostic approaches in non‐expert and expert laboratories for different clinical scenarios and discuss the role of genetic testing in the porphyrias. To diagnose porphyria in a currently symptomatic patient requires analysis of biochemical markers to demonstrate typical patterns of haem precursors in urine, faeces and blood. The use of genomic sequencing in diagnostic pathways for porphyrias requires careful consideration, and the demonstration of increased porphyrin‐related markers is necessary prior to genomic testing in symptomatic patients. In the acute porphyrias, genomic testing is presently a useful adjunct for genetic counselling of asymptomatic family members and the most common cutaneous porphyria, porphyria cutanea tarda, is usually a sporadic, non‐hereditary disease. Getting a correct and timely porphyria diagnosis is essential for delivering appropriate care and ensuring best patient outcome.

**Abbreviations**
AIP: acute intermittent porphyria
ALA: δ‐aminolevulinic acid
ALAD: δ‐aminolevulinic acid dehydratase
CEP: congenital erythropoietic porphyria
EPP: erythropoietic protoporphyria
HCP: hereditary coproporphyria
HEP: hepatoerythropoietic porphyria
HGMD: human gene mutation database
HPLC: high performance liquid chromatography
Ipnet: International porphyria network
PBG: porphobilinogen
PCT: porphyria cutanea tarda
VP: variegate porphyria
XLEPP: X‐linked erythropoietic protoporphyria

**Key points**

- The porphyrias are a group of rare inborn errors of metabolism associated with various clinical presentations and long‐term complications.
- Clinical features alone are not specific enough to establish a porphyria diagnosis.
- Diagnosis in a symptomatic patient depends on demonstration of typical patterns of haem precursors, for most diagnoses in urine, faeces and blood.
- Secondary abnormalities of porphyrin accumulation and excretion are more frequently occurring than the porphyrias.
- Genomic testing should not be used for diagnostic screening in a symptomatic patient without prior biochemical testing having demonstrated increased porphyria‐related diagnostic markers.

## 1 INTRODUCTION

The porphyrias are a group of rare inborn errors of metabolism caused by abnormal functioning of haem biosynthesis enzymes and are associated with various clinical presentations. These range from acute neurovisceral attacks characterized by severe abdominal pain and neuropsychiatric symptoms that may require highly specialized intensive care to chronic skin symptoms in the form of bullae on sun‐exposed areas or acute painful photosensitivity (Table 1).^1^ In the erythroid porphyrias, symptoms may be evident in newborns, as is often the case for congenital erythropoietic porphyria (CEP), or in children, most frequently erythropoietic protoporphyria (EPP). Acute intermittent porphyria (AIP), variegate porphyria (VP) and hereditary coproporphyria (HCP) usually become symptomatic in early adulthood, whereas porphyria cutanea tarda (PCT) most often presents in adults or older age. Additionally, depending on the type of porphyria, they may be associated with various long‐term complications such as liver disease including primary liver cancer and acute liver failure, chronic kidney disease and renal failure, hypertension, osteoporosis, vitamin D‐deficiency and anaemia. The wide array of clinical presentations at different stages of life highlights the porphyrias as relevant for many different clinical specialities. All, but one form of porphyria disease, are hereditary, with inheritance patterns including autosomal dominant, autosomal recessive and X‐linked inheritance. However, despite the increasingly important role of genetic testing in rare diseases today, this is of limited value in patients with symptomatic porphyrias. The autosomal dominant porphyrias have low clinical penetrance and likely pathogenic variants are frequently observed in the general population,^2^ without being associated with metabolic or clinical disease. Furthermore, the most common porphyria, PCT, is in most populations a sporadic disease in the majority of patients.^3^, ^4^, ^5^, ^6^

**TABLE 1:** An overview of the different porphyrias, with gene‐related information and main clinical presentation, presented in the order of the haem biosynthesis enzymes.

Disorder	Inheritance	OMIM	Genes	Chromosome	Gene size (~kb)	Expression	Other implicated genes	Main clinical presentation
X‐linked erythropoietic protoporphyria (XLEPP)	XL	300752	*ALAS2*	Xp11.21	22	Erythroid cells	*CLPX*	Acute painful photosensitivity
ALA‐dehydratase deficiency porphyria	AR	612740	*ALAD*	9q32	15	Ubiquitous and erythroid‐specific mRNAs		Abdominal pain Neurological symptoms
Acute intermittent porphyria (AIP)	AD	176000	*HMBS*	11q23.3	9	Ubiquitous and erythroid‐specific isoenzymes		Abdominal pain Neurological symptoms
Congenital erythropoietic porphyria (CEP)	AR	263700	*UROS*	10q26.2	38	Ubiquitous and erythroid‐specific mRNAs	*GATA1*	Severe bullous skin lesions Haemolytic anaemia
Porphyria cutanea tarda (PCT)	AD/sporadic	176100	*UROD*	1p34.1	3	Ubiquitous		Bullous skin lesions
Hereditary coproporphyria (HCP)	AD	121300	*CPOX*	3q11.2	14	Ubiquitous		Bullous skin lesions Abdominal pain Neurological symptoms
Variegate porphyria (VP)	AD	176200	*PPOX*	1q23.3	5	Ubiquitous		Bullous skin lesions Abdominal pain Neurological symptoms
Erythropoietic protoporphyria (EPP)	AR	177000	*FECH*	18q21.31	42	Ubiquitous		Acute painful photosensitivity

Abbreviations: AD, autosomal dominant; AR, autosomal recessive; XL, X‐linked.

In a patient with symptoms consistent with a porphyria disorder, the definitive diagnosis depends on the demonstration of increased accumulation and excretion of porphyrins and porphyrin precursors, for most diagnoses in urine, faeces and blood (Table 2).^1^ Importantly, when assessing such a patient, it is necessary not only to be able to diagnose that the patient has a porphyria disease, but also to differentiate between the different porphyrias which may have overlapping symptoms and biochemical alterations. Discussion with an expert centre or porphyria specialist laboratory may be helpful. The lack of necessary pre‐analytical precautions, inadequate requesting of porphyria‐related markers, poor analytical quality and inappropriate interpretation of test results may all lead to an incorrect diagnosis being made.^7^ The full spectrum of porphyria diagnostics is typically performed by expert laboratories, whereas many other laboratories may perform a few porphyria‐related diagnostic tests. For a non‐expert laboratory, it is essential that the correct first‐line diagnostic markers are performed to rule in or rule out a porphyria diagnosis and that the laboratory is aware when to recommend further analysis at an expert laboratory to ensure correct diagnostics. Where incomplete testing exists, caution must be exercised in interpretation towards a porphyria diagnosis. If possible, repeat or further testing should be requested from, or in collaboration with, a competent expert/specialist laboratory, and clinical symptoms must be carefully considered together with the biochemical data. The International Porphyria Network (Ipnet) Laboratory Working Group^8^ aims to improve the quality of porphyria diagnostics worldwide, including developing guidelines and recommendations for diagnosing and monitoring. In this study, we provide an overview of porphyria‐related tests with their strengths and limitations, give recommendations on requesting and diagnostic approaches for different clinical scenarios and discuss the role of genetic testing in the porphyrias.

**TABLE 2:** Overview of typical biochemical findings in urine, faeces, erythrocytes and plasma for the different porphyrias.

Porphyria disorder	Urine/plasma ALA and PBG	Urinary porphyrins	Faecal porphyrins	Erythrocyte porphyrins	Plasma fluorescence emission scan (peak; nm)
ALA‐dehydratase deficiency	ALA	Copro III	–	Zn proto	–
Acute intermittent porphyria (AIP)	PBG, ALA	Uro^b^	(Uro)	–	Negative/615–622
Hereditary coproporphyria (HCP)	PBG, ALA^a^	Copro III Uro^b^	Copro III Increased CIII:I	–	Negative/615–622
Variegate porphyria (VP)	PBG, ALA^a^	Copro III Uro^b^	Proto > copro Increased CIII:I	–	624–628
Congenital erythropoietic porphyria (CEP)	–	Uro I Copro I	Copro I	Uro I, copro I, Zn proto	615–618
Porphyria cutanea tarda (PCT)	–	Uro I/III Hepta	Isocopro, hepta, penta	–	615–618
Hepatoerythropoietic porphyria (HEP)	–	Uro I/III Hepta	Isocopro, hepta, penta	Zn proto	615–618
FECH‐deficient erythropoietic protoporphyria (EPP)	–	–	(Proto)	Metal‐free proto	626–634
X‐linked erythropoietic protoporphyria (XLEPP)	–	–	(Proto)	Metal‐free proto Zn proto	626–634

Abbreviations: ALA, δ‐aminolevulinic acid; CIII:I, coproporphyrin isomer III:I ratio; PBG, porphobilinogen.

^a^ In ongoing current acute attack.

^b^ Conversion from PBG.

## 2 PORPHYRIA‐RELATED BIOCHEMICAL DIAGNOSTIC TESTS

When selecting diagnostic strategies for the porphyrias, there are different approaches that can be taken, as to the type of diagnostic markers used. Porphyria diagnostics may include the quantification of porphyrins and porphyrin precursors in urine, faeces and blood, analysis of haem biosynthesis enzyme activity and gene sequencing, gene dosage and other genetic tests of haem biosynthesis genes and other genes implicated in the porphyrias. Biochemical analysis is the recommended first‐line diagnostic approach in a symptomatic patient and may include quantification of (i) delta‐aminolevulinic acid (ALA) and porphobilinogen (PBG) in urine and/or plasma, (ii) total porphyrins and fractionation of individual porphyrins with clinically relevant porphyrin isomers in urine, faeces, plasma and erythrocytes and (iii) measurement of haem biosynthesis enzymes. In Table 3, all clinically relevant biochemical porphyria‐related analytes are listed with information on diagnostic and monitoring utility, preferred methodology with reference limits and recommended units, comments and supporting references.

**TABLE 3:** Key diagnostic utility, use in monitoring and non‐porphyria settings, preferred analytical methods with reference limits and interpretation of porphyria‐related markers in urine, erythrocytes, plasma and faeces.

Parameter	Key diagnostic utility	Use in monitoring/follow‐up	Non‐porphyria setting	Preferred methodology	Reference limits	Units	Comments	References
*Urine*								
ALA	Diagnosis of acute porphyria attack	Monitoring of acute attack Monitoring of remission or relapse	Increased after exposure to lead, or other toxins including alcohol. Increased in hereditary tyrosinemia	Cation exchange chromatography + colorimetry (Ehrlich) LC–MS	<5^9^ (cation exchange chromatography) <1.47^10^ <2.1^11^ (LC–MS)	μmol/mmol creatinine	Increased in acute porphyria attacks (AIP, VP, HCP, and ALAD deficiency porphyria). Usually analysed together with PBG Normal results do not exclude an acute porphyria in remission or a latent porphyria Increased over time in asymptomatic high excretors (AIP) Relatively stable in urine (<10% decrease after 7 days at room temperature) Colorimetric methods: false negative; methenamine hippurate, false positives; tienam, penicillin	[12] [13] [14] [15] [16] [17] [18] [19] [10] [11]
PBG	Diagnosis of acute porphyria attack (AIP, VP, HCP)	Monitoring of acute attack Monitoring of remission or relapse		Anion exchange chromatography + colorimetry (Ehrlich) LC–MS	<.8^9^ <1.45^20^ (anion exchange chromatography) <.137^10^ <.2^11^ (LC–MS)	μmol/mmol creatinine	Highly specific diagnostic marker of acute porphyria attack of AIP, VP, HCP. Normal/marginally increased in ALAD deficiency porphyria Normal results do not exclude an acute porphyria in remission or latent porphyria Increased over time in asymptomatic high excretors (AIP) Unstable in urine due to formation of uroporphyrin & glycine conjugates Colorimetric methods: false negative; methenamine hippurate, false positives; tienam	[21] [22] [23] [24] [25] [19] [16] [9] [20] [10] [11]
Total porphyrins	PCT, HEP, CEP diagnosis	Monitoring of treatment, remission, and relapse for PCT	Secondary coproporphyrinuria (alcohol, environmental chemicals, liver, haematological and infectious diseases, toxins, drugs, for example sex hormones and anaesthesia‐related, pregnancy, fasting, myocardial infarction, hereditary hyperbilirubinaemia, diabetes mellitus, malignant disorders)	Fluorometry Spectrophotometry HPLC of acidified urine	<35^26^	nmol/mmol creatinine	Increased in PCT, HEP, CEP. May be marginally increased in VP & HCP. AIP: may be increased if increased PBG Fluorescence measured in very diluted urine may lead to misleading results Fluorescence emission wavelength maximum varies depending on the predominance of uroporphyrin or coproporphyrin Normal in pseudoporphyria (bullous skin symptoms but normal porphyrin metabolism) induced by drugs (NSAIDs, antibiotics, diuretics) or ultraviolet light	[27] [28] [29] [30] [31] [26] [32] [33] [34] [35] [36]
Uroporphyrin I	PCT, HEP, CEP diagnosis	Monitoring of treatment, remission, or relapse for PCT		HPLC	<3.9^9^	nmol/mmol creatinine	Increased in PCT/HEP & CEP Increased in AIP if increased PBG Spontaneous formation in urine from condensation of PBG	[27] [9]
Uroporphyrin III	PCT, HEP diagnosis			HPLC	<2^9^	nmol/mmol creatinine	Increased in PCT/HEP Normal in CEP AIP: may be increased if increased PBG Physiological formation uncertain	[35] [9] [37]
Heptacarboxyl porphyrin	PCT, HEP diagnosis			HPLC	<1.3^9^	nmol/mmol creatinine	Isomer increase highly specific of PCT/HEP Normal in CEP	[35] [9]
Total coproporphyrins			Secondary coproporphyrinuria See above	HPLC	<22^38^	nmol/mmol creatinine		[38]
Coproporphyrin I	CEP diagnosis	(Monitoring of EPP liver disease)	Hereditary hyperbilirubinaemias, cholestatic jaundice, hepatitis, and cirrhosis	HPLC	<8.5^9^	nmol/mmol creatinine	Highly increased in CEP May be increased in cholestatic liver disease (EPP) Increased in hereditary hyperbilirubinaemias: Dubin–Johnson, Rotor & Gilbert's Syndromes (increased copro I:III ratio)	[39] [40] [41] [42] [9]
Coproporphyrin III			Secondary coproporphyrinuria see above	HPLC	<26^9^	nmol/mmol creatinine	Increased in lead and other heavy metal intoxications Increased in AIP & ALAD deficiency porphyria	[43] [44] [9]
*Erythrocytes*								
Total porphyrins	Erythropoietic porphyria diagnosis EPP, XLEPP and CEP	Monitoring of EPP, XLEPP & CEP	Increased in iron deficiency, some other forms of anaemia, and lead intoxication	Fluorometry or HPLC	.4–1.7	μmol/L erythrocytes	Increased in EPP, XLEPP, CEP and all homozygous porphyrias	[29]
Total protoporphyrin	EPP, XLEPP diagnosis	Monitoring of EPP & XLEPP	Iron deficiency anaemia and lead intoxication	Fluorometry or HPLC	.4–1^9^ <1.5^45^	μmol/L erythrocytes	Increased in EPP & XLEPP	[46] [9] [45]
Metal‐free protoporphyrin	EPP, XLEPP diagnosis			Fluorometry (emission peak 630 nm) or HPLC	<15^47^	% of total protoporphyrin	EPP: only metal‐free protoporphyrin increased (>80% of total protoporphyrin) XLEPP: both metal‐free and zinc chelated protoporphyrin increased	[48] [29] [47]
Zinc protoporphyrin	EPP, XLEPP diagnosis		Iron deficiency anaemia and lead exposure	Fluorometry (emission peak 587 nm) or HPLC	<.9^49^	μmol/L erythrocytes	Increased in XLEPP Increased in HEP, CEP and all homozygous porphyrias May be calculated as % of total protoporphyrin	[50] [51] [52] [53] [49] [29]
Uroporphyrin	CEP diagnosis	CEP monitoring		HPLC	<1	nmol/L erythrocytes	Increased in severe CEP; biomarker of disease severity	[54]
PBG deaminase activity	(AIP diagnosis/ family investigations; if genetic analysis is not available or inconclusive)			Uroporphyrin formation after incubation of hemolysates with PBG	6.1–20^20^	nmol·s^−1^·L^−1^	Normal in non‐erythroid forms of AIP Influenced by haematopoietic status Overlap between activities in *HMBS* variant carriers and healthy; genetic analysis of *HMBS* is recommended May be increased if sampling performed when in an acute attack	[55] [56] [57] [58] [20]
UROD activity	(Familial PCT/HEP diagnosis/family investigations if genetics not available)			Coproporphyrin formation after incubation of hemolysates with pentacarboxyl‐porphyrinogen	1.8–4^59^	μmol coproporphyrin/min/L erythrocytes	Overlapping of results between familial and sporadic cases; genetic analysis of *UROD* is recommended	[60] [59] [61]
*Plasma*								
Plasma fluorescence peak	VP diagnosis Diagnosis & classification of porphyria		Small increase in end stage renal failure	Fluorescence emission spectroscopy of diluted plasma	Negative	Positive/negative fluorescence emission peak after excitation (405 nm)	Highly sensitive and specific front‐line test Emission maximum wavelengths differ according to the type of porphyria Diagnostic for symptomatic VP (624–628 nm)	[62] [63] [64]
Total porphyrins	Diagnosis of cutaneous porphyria	Monitoring of treatment, remission or relapse for PCT	Possible increase in renal disease	Fluorometry HPLC	<19^54^	nmol/L	Increased in PCT & CEP Historical studies report moderately increased in patients with renal failure Not increased in pseudoporphyria	[65] [66] [54]
Uroporphyrin	Diagnosis of cutaneous porphyria PCT, HEP, CEP			HPLC	<1^9^	nmol/L	PCT‐type increases have been reported in historical studies of patients with renal disease, but recent case‐series with improved haemodialysis techniques indicate low prevalence	[9] [66] [67]
Heptacarboxyl porphyrin	PCT, HEP diagnosis Diagnosis of cutaneous porphyria			HPLC	<5^9^	nmol/L	Increased in PCT/HEP In renal failure; uro/7‐carboxyl porphyrin ratio may be higher than in PCT	[66] [9]
ALA	Diagnosis of acute porphyria attack	Acute attack monitoring, especially in kidney failure Response to treatment		LC/MS	<.41^68^ <.13^11^	μmol/L	Utility in anuric patients or acute porphyria patients with chronic kidney disease	[68] [11]
PBG	Diagnosis of acute porphyria attack	Acute attack monitoring, especially in kidney failure Response to treatment		LC/MS	<.12^68^ <.05^11^	μmol/L	Correlated with u‐PBG but possibly stronger association with symptoms Utility in anuric patients or acute porphyria patients with severe chronic kidney disease	[68] [69] [11]
*Faeces*								
Total porphyrins	Diagnosis and classification of porphyria		Diet, gastrointestinal haemorrhage	Spectrophotometry or Fluorometry or HPLC	<72^9^ <192^20^	nmol/g dry weight	Increased in PCT, HEP, CEP, VP, HCP, EPP & XLEPP Non‐specific increases of dicarboxylic porphyrins due to bacterial degradation of haem in the gut, the haem may be dietary in origin or pathological (gastrointestinal bleeding)	[9] [20] [29]
Total coproporphyrin	Diagnosis and classification of porphyria			Spectrophotometry or Fluorometry or HPLC	14–45	nmol/g dry weight	Specially increased in CEP, VP & HCP	[45]
Coproporphyrin III:I ratio	HCP diagnosis Diagnosis and classification of porphyria (Family investigation for HCP if genetics unavailable)			HPLC	.63^9^ .3–1.4^20^ .1–1.2^a^	–	Increased ratio is main diagnostic marker for symptomatic HCP. Also often increased in VP In CEP: lower ratio than in healthy individuals	[9] [20]
Isocoproporphyrin	PCT, HEP diagnosis	Monitoring of PCT remission		HPLC	<1	nmol/g dry weight	Increased in PCT/HEP with co‐increase of desethylisocoproporphyrin Non‐specific small increases also in other porphyrias, dependent on methodology	[7] [70]
Mesoporphyrin				HPLC		–	Co‐increased with protoporphyrin in EPP	[71]
Harderoporphyrin	HCP diagnosis and disease classification			HPLC	Not detected^72^	–	Highly increased in rare form of homozygous coproporphyria (harderoporphyria)	[73] [72]
Protoporphyrin		(Monitoring of EPP & XLEPP)		HPLC	<38^9^ 33–170^45^	nmol/g dry weight	Increased in VP and EPP/XLEPP. A decrease from baseline in EPP patients may indicate intrahepatic cholestasis	[9] [45]

Abbreviations: AIP, acute intermittent porphyria; ALA, δ‐aminolevulinic acid; ALAD, ALA dehydratase; CEP, congenital erythropoietic porphyria; EPP, erythropoietic protoporphyria; HCP, hereditary coproporphyria; HEP; hepatoerythropoietic porphyria; HPLC, high‐performance liquid chromatography; LC/MS, liquid chromatography coupled to mass spectrometry; PBG, porphobilinogen; PCT, porphyria cutanea tarda; UROD, uroporphyrinogen decarboxylase; VP, variegate porphyria; XLEPP, X‐linked erythropoietic protoporphyria.

^a^ Derived from HPLC analysis of normal faeces (*n* = 275) in the Hospital Clinic of Barcelona (2020–2022) (unpublished data).

**FIGURE 1:**
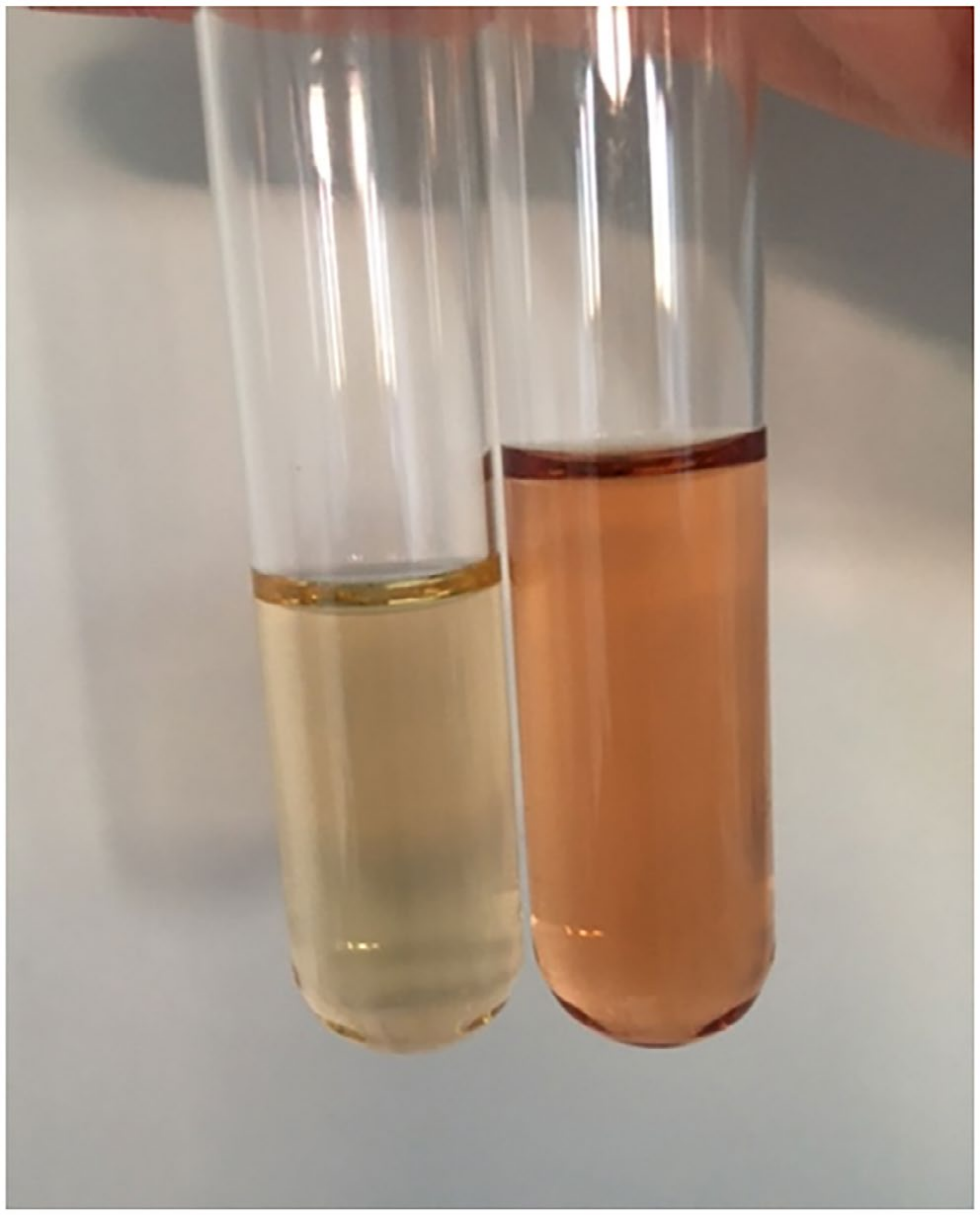
Urine colour during an acute attack in a patient with acute intermittent porphyria. When the sample was taken, the urine colour was normal (left), whereas a sample of the same urine exposed 1 hour under a heating lamp (right) revealed the typical red‐brown colour (ALA: 36 μmol/mmol creatinine; PBG: 110 μmol/mmol creatinine, urine creatinine: 3.5 mmol/L).

**FIGURE 2:**
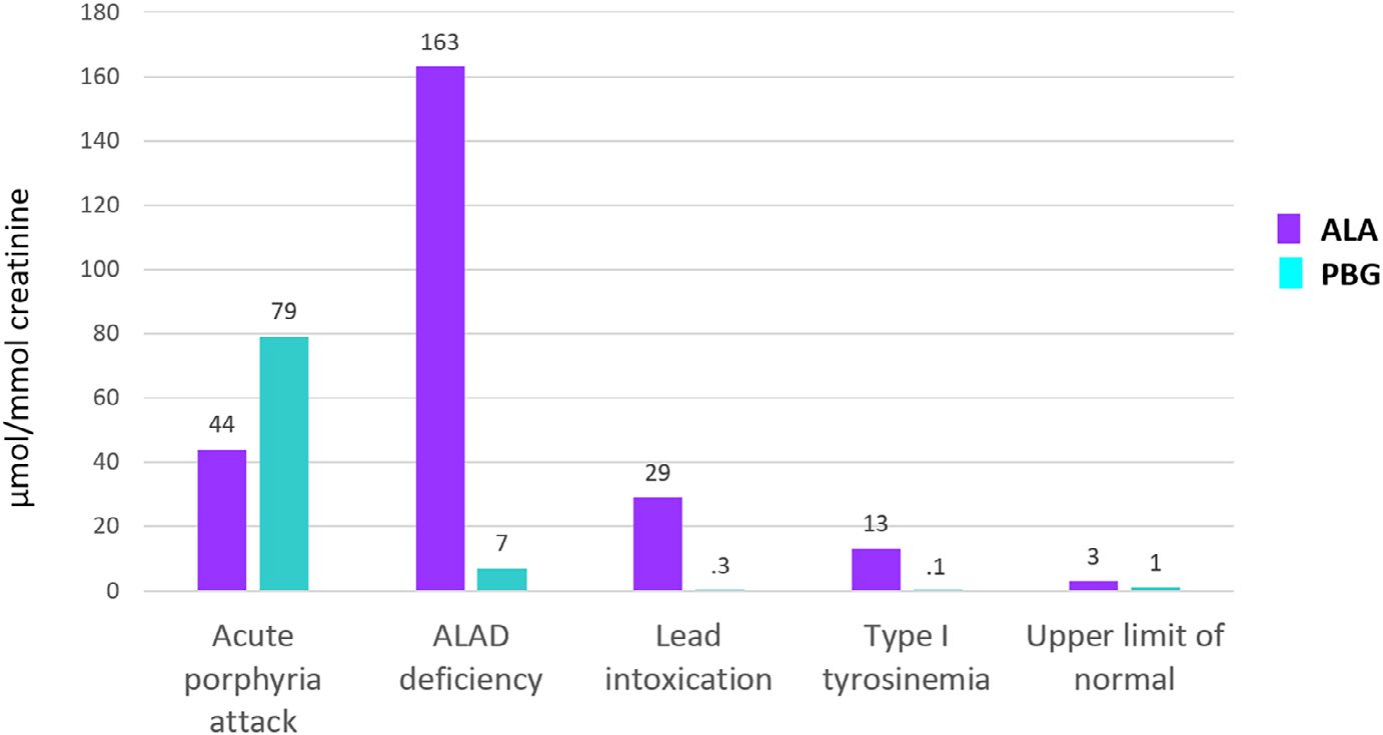
ALA and PBG concentrations in samples from a patient with acute intermittent porphyria when in an acute attack, ALAD deficiency porphyria, lead intoxication, and tyrosinemia type 1. Upper limits of normal for ALA and PBG are also indicated. Data from ALAD deficiency porphyria were derived from Thunell, S. et al. “Aminolaevulinate dehydratase porphyria in infancy. A clinical and biochemical study.” J Clin Chem Clin Biochem. (1987). The patients with acute lead poisoning and type I tyrosinemia were symptomatic with abdominal pain when sampled.

Generally, most porphyria‐related markers cannot be interpreted on an individual basis to reach a correct diagnosis, as different diseases may cause accumulation of the same metabolites (Table 3). However, there are first‐line markers that can be used to rule in or rule out porphyria as a potential cause of current symptoms in a patient. For a patient presenting with current acute neurovisceral symptoms, these include urinary/plasma ALA and PBG, which if results are normal, can be used to rule out an acute porphyria, that is ALA dehydratase (ALAD) deficiency porphyria, VP, HCP and AIP (Table 1), when all conditions for proper sample collection and analysis are met. If, on the other hand, PBG is significantly increased, a diagnosis of acute porphyria can be made^74^ (Figure 1). ALA is less specific than PBG and urinary ALA can also be significantly increased in lead intoxication and hereditary tyrosinemia (Figure 2), which may present with similar symptoms as the acute porphyrias.^75^, ^76^ Historically, methods for ALA and PBG have been based on ion exchange chromatography, but today mass spectrometry methods are becoming more common.^10^, ^11^, ^77^ When interpreting results, it is important to be aware that mass spectrometry methods are more sensitive and associated with lower limits of normal, than traditional ion‐exchange methods.^10^, ^11^ When investigating a patient with symptoms raising suspicion of an acute porphyria, including urinary total porphyrins as a first‐line analysis is unhelpful and may be misleading. In the acute porphyrias, urinary total porphyrin concentrations may be increased, mainly or in part due to in vitro polymerization of PBG to uroporphyrin.^9^ However, increases, mainly of coproporphyrin, occur frequently in many other conditions such as hepatobiliary disease, alcohol use and other common disorders (Table 3).^31^, ^36^

Plasma fluorescence scanning, that is fluorescence emission spectroscopy of diluted plasma with excitation at 405 nm, can be used to rule in or rule out porphyrias presenting with bullous symptoms, that is CEP, VP, HCP and PCT and the “homozygous” form of PCT, hepatoerythropoietic porphyria (HEP).^62^, ^63^ The emission maximum wavelength differs according to the type of porphyria (Table 2). Analysis of urinary total porphyrins is not recommended as the single front‐line rule‐in test in a cutaneous setting, as VP patients with current cutaneous symptoms can have normal concentrations of urinary porphyrins,^78^ as will EPP. In addition, secondary coproporphyrinuria will more frequently be the cause of an increased urinary total porphyrins result (Table 3), than a porphyria disorder. Thus, increased urinary total porphyrins must be followed by urinary porphyrin fractionation of individual porphyrins. Estimation of urinary total porphyrins with fractionation of individual porphyrins is important for the diagnosis of PCT, for monitoring of PCT during treatment and for the assessment of a PCT relapse. The quantification of different types of porphyrin isomers in urine is also required for the definitive diagnosis of CEP,^40^ as well as for investigation of the non‐porphyria‐related disorders hereditary hyperbilirubinaemias, Dubin–Johnson, Rotor and Gilbert's syndromes (Table 3).^31^, ^41^ Urinary total porphyrins are, in routine practice, measured by methods such as fluorometry, spectrophotometry and high‐performance liquid chromatography (HPLC), and commercial methods are available. For fractionation, HPLC is most commonly used.^7^

Faecal total porphyrins may be increased in most of the cutaneous porphyrias including PCT, HEP, CEP, VP, HCP, EPP and X‐linked erythropoietic protoporphyria (XLEPP, known as XLP and XLDP in some publications),^9^, ^20^, ^64^ but can also be increased due to bacterial degradation of haem, either dietary in origin or pathological due to gastrointestinal bleeding, in the gut (Table 3). Fractionation of porphyrins in faeces is of particular importance for the diagnosis and differentiation of HCP, CEP and PCT/HEP. Quantification of protoporphyrin in erythrocytes is necessary to establish the diagnosis of EPP and XLEPP in a patient with acute painful photosensitivity symptoms.^53^, ^79^ It is also of diagnostic value for CEP and all other homozygous porphyrias, in which increased zinc‐chelated protoporphyrin is typically observed. Erythrocyte zinc‐chelated protoporphyrin also increases in iron deficiency and lead intoxication.^49^, ^50^

Measurement of enzyme activity plays a limited role in porphyria diagnostics today, following the introduction of genetic tests. PBG deaminase and, to a lesser extent, uroporphyrinogen decarboxylase are the enzymes mostly commonly examined, as they can be measured in red blood cells. They may be used to assess patients with a biochemical porphyria diagnosis when genetic analysis is not available or in patients in whom genetic analysis does not identify the expected pathogenic variant(s). It is, however, important to be aware that there is quite a big overlap in measured enzyme activity in erythrocytes between healthy persons and patients with porphyria.^20^, ^61^

## 3 PRE‐ANALYTICAL REQUIREMENTS AND REQUESTING OF PORPHYRIA‐RELATED BIOCHEMICAL TESTS

Porphyrins are sensitive to light, and it is recommended that all samples are protected from light to avoid falsely low or negative results. This can be achieved by covering sampling tubes in aluminium foil. Though there is generally a lack of robust stability data for porphyrin markers, it is well established that PBG starts to decrease within 24 hours when kept at room temperature.^11^, ^80^ This is of particular relevance for hospitals which do not perform quantitative PBG analysis and which will send samples by post or courier to another laboratory for analysis. Urine samples should be analysed within 24 hours of sampling or should be kept at 4 degrees or frozen prior to analysis. However, PBG is also sensitive to repeated freeze‐thawing cycles.^81^ ALA, on the other hand, is more stable at room temperature.^11^, ^80^ A morning or spot urine sample should be collected for the analysis of urinary markers, and urinary results should be normalized to the excretion of creatinine.^82^ The results of samples where urinary creatinine is below 2 mmol/L should be interpreted with caution, as these can appear falsely elevated due to the low creatinine concentration. Twenty‐four hour collections are not recommended.

Relevant clinical information is essential to determine which biochemical tests are necessary to diagnose or exclude likely diagnoses and to correctly interpret the results. For rare disorders like the porphyrias, most physicians cannot be expected to know which are the required markers for the different clinical scenarios. Thus, specialist laboratories are recommended to provide a dedicated request form for porphyria investigations. This form should provide information on required pre‐analytical treatment of samples and include fields for relevant clinical information, preferably as a list of symptoms from which to select. It is also essential to know if the patient is currently symptomatic, or if not, the date of presentation of symptoms. For a patient with a positive family history, information on the type of porphyria, the relationship to the proband and the genetic variant, if known, should be given.

## 4 CLINICAL SCENARIO: ACUTE NEUROVISCERAL SYMPTOMS

The three autosomal dominant acute porphyrias, AIP, VP and HCP, may present with acute neurovisceral attacks. A consensus among international experts, based on the Delphi method, recently agreed on a definition for an acute porphyria attack.^74^ This defines an acute porphyria attack as an episode with defined clinical manifestations persisting for at least 24 hours in the absence of other likely explanation and significantly increased urinary PBG/creatinine ratio. During an attack, the PBG/creatinine ratio is typically increased to more than 10 times the upper limit of normal when analysed by the most frequently available analytical methods, including commercial assays. If measured by a mass spectrometry method which usually has lower limits of detection and correspondingly lower upper limits of normal, a result above 10 μmol/mmol creatinine is expected.^11^ In AIP patients, PBG concentration typically remains elevated for years after an acute episode.^83^ Thus, if the patient's PBG when asymptomatic is raised, a significant further increase above baseline is expected during attacks. There is, however, limited evidence demonstrating the size of such an increase. If a PBG value from a recent baseline sample is not available, assessment of a patient with a known AIP diagnosis may depend on clinical evaluation of symptoms and findings, as detailed in the recent Ipnet recommendation.^74^ If urinary PBG excretion is normal in a patient with current symptoms, acute porphyria is excluded as the cause, if the sample has been correctly treated. The exception is the extremely rare autosomal recessive ALAD deficiency porphyria, where PBG typically is normal or near normal, but where ALA and coproporphyrin III are significantly increased^17^ (Table 3). In an acute porphyria patient treated with hemin, the standard treatment for an acute attack, PBG excretion may be lower or normalized if sampling is performed during or shortly after hemin treatment. Screening tests to detect the presence of PBG in urine are generally not recommended due to low specificity and sensitivity. If, however, such screening tests are still used, for example in an emergency room setting in hospitals where quantitative assays are not available, results must be confirmed by a quantitative and sensitive assay to ensure an appropriate diagnostic process.

In order to biochemically differentiate between the three autosomal dominant porphyrias in a patient with current acute symptoms, analysis of faecal coproporphyrin III:I ratio and plasma fluorescence scanning are required.^20^ A clear plasma fluorescence peak at 624–628 nm establishes the diagnosis of VP.^20^, ^64^ However, a normal plasma fluorescence scan does not rule out AIP or HCP nor does a positive result distinguish AIP from HCP; in both conditions, an emission peak around 620 nm may be present. Anecdotally, two VP patients have been reported where the plasma fluorescence maximum had shifted into the lower range during an acute attack.^20^, ^64^ Faecal coproporphyrin III:I ratio is increased in symptomatic HCP and may also be increased in VP.^44^, ^84^ The diagnosis of HCP is therefore established based on the demonstration of an increased faecal coproporphyrin III:I ratio when VP has been excluded by the plasma fluorescence scan wavelength. For a patient with VP or HCP, PBG excretion may return to normal or near normal within a short time period after the cessation of symptoms and the attack has resolved. Thus, for a patient where symptoms are subsiding or the patient is no longer symptomatic, analysis of faecal coproporphyrin III:I ratio and plasma fluorescence scanning must also be performed when PBG is normal.^20^

In an individual known to have inherited a genetic predisposition for one of the acute porphyrias, normal PBG/ALA concentration excludes an acute attack as cause of current symptoms. A healthy individual in whom a pathogenic gene variant has been identified as part of family investigation is, according to the Ipnet definitions, defined as latent acute porphyria if he/she has never experienced definite manifestations of acute porphyria and the urine PBG/creatinine ratio is lower than 4 times the upper limit of normal.^74^ A subgroup of those with a pathogenic variant consistently have increased PBG/creatinine ratio over time, without ever experiencing an acute attack. These are termed asymptomatic high excreters if the urine PBG/creatinine ratio is at least 4 times the upper limit of normal. In such individuals, the attribution of symptoms to acute porphyria may largely depend on clinical assessment and an elevated PBG above the baseline concentration, taking into account the high natural biological variation of urinary PBG.^82^

Patients with acute porphyria have increased risk of renal failure, in particular AIP patients with a particular *PEPT2* gene variant, likely by a mechanism of ALA toxicity in renal cells.^85^ Chronic kidney disease is therefore a common feature in patients with symptomatic AIP.^86^, ^87^ Particular attention should be paid when assessing the urine of patients with a decreased renal function. It has been shown that in most clinical situations, urine and plasma concentrations are relatively well correlated for both ALA and PBG.^11^ The study by Poli et al., however, demonstrates that a decrease in estimated glomerular filtration rate alters the excretion of ALA and PBG and that there is an underestimation of haem precursor levels in acute porphyria patients with chronic kidney disease, when measured in urine.^11^ When possible, it is recommended to monitor these patients with plasma ALA and PBG, especially when an acute attack is suspected. The PBG/ALA ratio in urine is around 2 in AIP patients with normal renal function, but this ratio increases in parallel to the impairment of the glomerular filtration.^88^, ^89^ Consequently, in AIP patients with severe chronic kidney disease, a relatively low ALA concentration compared to PBG is observed in urine.

## 5 CLINICAL SCENARIO: CUTANEOUS SYMPTOMS

The cutaneous porphyrias are characterized by the accumulation of phototoxic porphyrins in the skin.^90^ These can cause skin damage following sun exposure to light between 400 and 410 nm due to photoactivation of porphyrins and generation of singlet oxygen in the dermis. Cutaneous porphyrias can be divided into two groups dependent on the type of skin symptoms: (i) those that present with acute photosensitivity (EPP, XLEPP) and (ii) those that present mainly with bullae, fragility of the skin and/or scarring. This latter group includes PCT, HEP and CEP. In addition, VP and HCP which although they are acute porphyrias can also present with bullous skin symptoms. Active skin lesions in cutaneous porphyria are accompanied by excess circulating porphyrins produced in either the liver or bone marrow.^91^ Patients with current cutaneous symptoms can therefore be expected to have increased plasma porphyrins, which is the rationale for using plasma fluorescence scanning as a first‐line analysis. All the bullous porphyrias except VP are associated with similar emission maximum wavelengths (Table 2), and to differentiate between them fractionation of porphyrins in urine, faeces and erythrocytes is required. PCT is characterized by uro‐ and heptacarboxyl porphyrins in urine and hepta‐, penta‐ and isocoproporphyrins predominate in faeces (Table 2). Fractionation of porphyrins in urine is in most laboratories the standard approach when investigating a potential PCT diagnosis, but fractionation of porphyrins in plasma may facilitate diagnosis in anuric patients with kidney failure. HEP is characterized by similar urinary, faecal and plasma porphyrin patterns as PCT^92^, ^93^ (Table 2), though porphyrin concentrations may be higher. In addition, erythrocyte zinc‐chelated protoporphyrin is typically increased in HEP, but confirmation of the diagnosis will usually require genetic analysis of the *UROD* gene. CEP, which is caused by deficiency of uroporphyrinogen synthase, is characterized by increased concentration of porphyrins of the isomer I type in urine, faeces, plasma and erythrocytes (Table 2).

For a patient with acute painful photosensitivity symptoms caused by EPP or XLEPP, plasma fluorescence is typically either around 628 nm if bound to globin or at 632–636 nm if bound to albumin.^94^, ^95^ In such a patient, the measurement of erythrocyte protoporphyrin with fractionation of metal‐free and zinc‐chelated protoporphyrin is essential to establish the diagnosis. It must be clarified if an increase in erythrocyte total protoporphyrin is due to metal‐free protoporphyrin as in EPP or to both metal‐free and zinc protoporphyrin as in XLEPP (Tables 2 and 3). In many cases, this may require genetic analysis of the *FECH* and *ALAS2* genes to ensure appropriate differentiation between FECH‐related EPP and X‐linked EPP, where the latter is caused by *ALAS2* gain‐of‐function variants.^53^

## 6 THE ROLE OF GENETIC TESTING IN THE PORPHYRIAS

When discussing the role of genetic testing in diagnosing the porphyrias, inheritance patterns, clinical penetrance and disease severity must be taken into account. Genes for all the porphyrias have been characterized, with some porphyrias also being caused by variants in genes not coding for enzymes in haem biosynthesis (Table 1). All porphyrias show extensive allelic heterogeneity and in most populations, variants are restricted to one or a few families, with the exception of a few founder effects.

### 6.1 Genomic testing in the acute porphyrias

The prevalence of pathogenic variants in the *HMBS* gene that cause a predisposition for AIP has been estimated at more than 1 in 2000 in the general population.^2^, ^96^ In some populations, founder effects produce higher prevalence of AIP, including the p.Trp198Ter variant in Sweden^97^ and p.Trp283Ter in Switzerland.^98^ The prevalence of overt AIP is estimated at 1 in ~200 000.^99^ This suggests a low penetrance in the general population of approximately 1%. VP is less common than AIP, but has a higher prevalence in South Africa, due to a founder effect (p.Arg59Trp).^100^ There is little information available for the penetrance of VP and HCP. In a South African family, 28 members were identified with the p.Arg59Trp variant but only one had overt VP.^101^ Using the population database GnomAD^102^ and pathogenic *PPOX* variants recorded in the Human Gene Mutation Database (HGMD) in 2022,^103^ a very rough estimate of individuals with a predisposition of VP can be made of 1 in 3000. The prevalence of overt VP has been calculated as 1 in 300 000,^99^ giving a similar 1% penetrance as is found in AIP. The penetrance of overt HCP is not known. In an Australian family of 22 family members with low coproporphyrinogen oxidase activity and raised faecal coproporphyrin III:I ratio, only one patient had a definite acute attack.^104^ The estimated prevalence of HCP in the UK is 1–2 in a million.^105^ Using the same approach as used for VP, a very rough estimate of individuals with a predisposition of HCP can be made of 1 in 4000, that is a penetrance figure of .4%. Although the evidence for penetrance is not very robust for VP and HCP, especially taking into account that not all variants reported in HGMD will necessarily meet the criteria for a pathogenic variant, it does indicate that penetrance is very low for all the autosomal dominant acute porphyrias.

The clinical symptoms of an acute porphyria attack may be non‐specific including symptoms such as abdominal pain, vomiting and nausea, constipation, or diarrhoea. If patients with these symptoms were investigated directly with genetic testing, without prior biochemical confirmation, patients with a pathogenic variant in the acute porphyria genes are likely to be discovered. This would likely lead to, from a clinical point of view, a “false” acute porphyria diagnosis, in patients who are predisposed for an acute porphyria, but where symptoms are not caused by the pathogenic variant as there is no metabolic activation of the disease. This may lead to incorrect treatment, potentially with serious consequences. Genome sequencing is a very effective technique, but if it is to be used for the diagnosis leading to treatment of acute porphyrias, it is essential that it can be demonstrated that the patient is currently experiencing an acute porphyria attack with increased accumulation of PBG, or in the case of ALAD deficiency porphyria, increased ALA. This means that patients with symptoms suggestive of acute porphyria should fulfil as a minimum the criteria for a currently ongoing acute attack, that is demonstrating markedly increased urine PBG/ALA, before performing relevant genetic investigations, sequencing and gene dosage of *HMBS*, *PPOX* and *CPOX*, or in the case of ALAD deficiency porphyria, *ALAD* (Table 4). If the minimum biochemical criteria have not been met and acute porphyria is still considered as the most likely diagnosis, fresh samples for biochemical analysis must be taken when the patient is symptomatic, ensuring that all pre‐analytical, storage, transport and analytical best practice is followed.

**TABLE 4:** Overview of required minimum biochemical criteria in a symptomatic patient prior to genetic testing and which genes to include in genomic testing for the different scenarios.

Symptomatic porphyria	Minimum biochemical criteria prior to genetic analysis	Genomic testing (sequencing and gene dosage)
ALAD deficiency porphyria	Increased urine/plasma ALA with exclusion of secondary causes	*ALAD*
Acute intermittent porphyria Variegate porphyria Hereditary coproporphyria	Increased urine/plasma PBG	*HMBS*, *PPOX*, *CPOX*
Porphyria cutanea tarda Hepatoerythropoietic porphyria Congenital erythropoietic porphyria Hereditary coproporphyria Variegate porphyria Erythropoietic protoporphyria X‐linked erythropoietic protoporphyria	Positive plasma porphyrin fluorescence scan Increased erythrocyte protoporphyrin	*UROD*, *UROS*, *GATA1*, *CPOX*, *PPOX, FECH*, *ALAS2*, *CLPX*

Abbreviations: ALA, δ‐aminolevulinic acid; PBG, porphobilinogen.

Studies in AIP families have shown that the likelihood of an acute attack is much higher in family members, estimated at 12.7% excluding the proband.^106^ Healthy at‐risk family members are therefore advised to have genetic testing so that those with a pathogenic gene variant can avoid potential precipitating factors and receive rapid treatment if symptomatic. Correctly identifying the causative variant in a family with an acute porphyria is therefore recommended. For the investigation of healthy at‐risk family members, testing for the family's acute porphyria pathogenic variant is the method of choice, as porphyria‐related biochemical markers may be normal in asymptomatic individuals.

### 6.2 Genomic testing in the cutaneous porphyrias

As with the acute porphyrias, the cutaneous symptoms associated with the porphyrias are not diagnostic in themselves. Genetic testing without prior biochemical analysis is therefore not advisable (Table 4). In addition to this, in PCT, which is the most common type of porphyria, only 20% have a pathogenic variant in the *UROD* gene (familial PCT), in most population for which data exist.^3^, ^4^, ^5^, ^6^ The remainder have sporadic PCT, which is not associated with *UROD* variants. Genomic testing can be undertaken in families where a pathogenic variant in the *UROD* gene has been identified in an index patient, and it may be useful for those at‐risk of blistering skin lesions to avoid known susceptibility factors. However, familial PCT has a low penetrance,^107^ so the likelihood of developing clinical manifestations is small and if they do occur, they can be treated. This makes predictive genetic testing for PCT optional. HEP is the “homozygous” variant of PCT. As compared to PCT, HEP is in most patients characterized by more severe photosensitivity symptoms from childhood and also by increased erythrocyte zinc‐chelated protoporphyrin.^108^ However, milder late‐onset cases may also appear. The diagnosis should be confirmed by the demonstration of two pathogenic *UROD* variants.

EPP has complex inheritance, and there are several different genetic mechanisms that cause the EPP phenotype. Most EPP cases are caused by a pathogenic variant on one *FECH* allele with the other containing an intronic variant (c.315‐48T>C) that causes reduced expression of the second allele, whereas approximately 4% of EPP patients have biallelic pathogenic variants. These two forms of EPP are thought to be completely penetrant.^109^ No evidence was found in 155 EPP families of any individuals with a genetic predisposition for EPP without symptoms. However, evidence from the United Kingdom Biobank^110^ showed a genetic prevalence of 1 in 20 000, while the calculated prevalence of overt EPP in the UK has been found to be 1 in 40 000.^99^ This discrepancy may be caused by underdiagnosis or possibly due to a lack of clinical recognition or mild symptoms. A third group of patients (XLEPP) with an EPP phenotype (2%–10%) has a gain‐of‐function variant in the final exon of the *ALAS2* gene. A single family has also been described with defects in both the *CLPX* and *ALAS2* genes,^111^, ^112^ and it is also likely that there are further mechanisms to be identified as pathogenic variants have not been identified in ~5% of patients with an EPP phenotype. Very rarely, cases of late onset EPP are observed, most commonly associated with myelodysplastic syndrome.^113^ In some cases, it has been shown to be due to an abnormality or somatic mutation in chromosome 18q (the locus of the *FECH* gene) trans to a low expression allele.^114^ Cytogenetic analysis and/or direct sequence analysis of bone marrow cells may be required to identify the cause.

## 7 DISCUSSION

The porphyrias may give rise to various clinical presentations and for some diagnoses, the symptoms may be the same as those associated with other far more common disorders. Thus, the porphyria diagnoses cannot be based on symptoms or clinical findings alone. To diagnose a symptomatic porphyria requires analysis of porphyria‐related biochemical markers to demonstrate typical patterns of haem precursors in urine, faeces and blood. Additionally, DNA‐analysis is sometimes warranted to differentiate between porphyrias with similar clinical presentation if biochemical findings are not definitive (e.g. FECH deficient and X‐linked EPP), to facilitate family investigations, especially for acute porphyrias where asymptomatic family members at risk are advised to have predictive genetic testing, and to inform on prognosis in CEP. Getting a correct and timely porphyria diagnosis is essential for appropriate care. However, this depends on a number of factors. Firstly, it requires the clinician to consider porphyria as a potential cause of the patient's symptoms. As a group of rare disorders which most physicians will never encounter, it may take time before the appropriate investigations are undertaken, as illustrated by reports on long diagnostic delay for example for EPP.^115^ It is also necessary that the correct sample materials are collected, transported and stored under appropriate conditions at the laboratory, together with clinical information on the patient's situation, to ensure the correct diagnosis is made. The physician or the laboratory must select an appropriate diagnostic strategy considering the clinical situation and the relevant biomarkers must be analysed with sufficiently sensitive and specific methods of high analytical quality. Finally, the results must be adequately interpreted taking into account the patient's clinical symptoms and results must be communicated, with interpretative comments, diagnosis and recommendations for further follow‐up, back to the clinician who then must act upon them. As is evident from this, there are many potential pitfalls before a correct diagnosis can be assured. Of note, results from the Ipnet External Quality Assessment Scheme which circulates native patient samples with clinical information to specialist laboratories worldwide have shown that most specialist laboratories report the correct diagnosis for patients with the most common diagnoses and where disease‐related markers are highly increased.^7^ However, the more unusual diagnoses or in cases with low or borderline concentrations of the diagnostic marker(s), the correct diagnosis may be overlooked. To assist laboratories in their diagnostic process, Ipnet best practice guidelines for diagnosis are currently under development and are expected to be available in 2024.

Non‐specialist laboratories may offer selected porphyria‐related tests, most usually qualitative screening tests or quantitative tests for urinary PBG and total porphyrins. Additionally, erythrocyte total protoporphyrin, which has historically been used as a marker for iron deficiency, may be available, but may, despite its name, not include metal‐free protoporphyrin.^52^ Presently, the quality of porphyria diagnostics services offered by non‐specialist laboratories is unclear. Furthermore, single raised results in isolation, especially increased urinary or faecal total porphyrins or coproporphyrin, can cause confusion. If the requesting physician or laboratory selects irrelevant tests for the clinical situation, this may also have severe impact on patient outcome. An example is analysing urinary total porphyrins for a patient with acute painful photosensitivity symptoms, where this analysis will provide no relevant information for a potential diagnosis of EPP or XLEPP. Another potential scenario is to utilize a qualitative screening test for PBG in urine in an emergency setting, without following this up with a quantitative and more sensitive assay. Further work to ensure that non‐specialist laboratories perform relevant first‐line screening test for the clinical situation, as detailed in this review, and that they provide information on when and where samples for more specialist investigation should be sent for analysis is necessary.

With the rapid development of genetic tests, some laboratories offer panels that include porphyria genes or include these in their diagnostic pathways. Genomic sequencing is a powerful and effective technique, but its use in diagnostic pathways for porphyrias requires careful consideration. Active porphyria is identified by biochemical methods and in acute porphyrias, genomic testing is most commonly used as useful adjunct for genetic counselling of asymptomatic family members. Regularly updated lists of genetic variants are available from the HGMD. However, not all the variants included in the HGMD have been confirmed as pathogenic. In addition, there are numerous variants identified by expert laboratories in patients with acute porphyrias not included in the HGMD. Ipnet is therefore currently working on establishing the Ipnet Variant Database.^116^ This aims to provide a quality‐assessed overview of pathogenic and relevant benign variants for the acute porphyria related genes, based on standardized biochemical criteria and appraisal by two independent experts using the American College of Medical Genetics guidelines.^117^ In the future, specialist centres will be able to submit their own variants for appraisal and then inclusion in the database. The availability of such a classification of pathogenicity of all identified acute porphyria gene variants will support accurate diagnosis.

Ipnet is a world‐wide association for specialist laboratories established in 2023. There is a lack of members from many countries around the world. In these countries, the state of porphyria diagnostic capabilities is unknown and likely not available. To help address this challenge, Ipnet has recently established the Acute Porphyria International Support Group. This aims to provide support to healthcare professionals looking after patients in countries where specialist care is not available. Their priorities are to facilitate diagnostic laboratory testing, management advice and better access to treatments. Further information on how to gain support from this group is available at Ipnet's website.^118^ These and other Ipnet initiatives such as the delivery of definitions in acute porphyrias^74^ and development of guidelines for diagnosis and treatment are important for porphyria patients receiving appropriate diagnostics and care, to ensure best patient outcome.

## AUTHOR CONTRIBUTIONS

All authors contributed to the study conception and design. Data collection was performed by Aasne K. Aarsand, Jordi To‐Figueras, Sharon Whatley and Caroline Schmitt. Aasne K. Aarsand wrote the first draft of the manuscript. All authors contributed to the interpretation of the results, critically revised the manuscript for important intellectual content, approved the final version to be published and agree to be accountable for all aspects of the work.

## CONFLICT OF INTEREST STATEMENT

AKA is an associate editor of Clinical Chemistry, and SS is part of the Data Monitoring Committee for a bitopertin phase 2 trial organized by Disc Medicine. The authors declare that they have no other known competing financial interests or personal relationships that could have appeared to influence the work reported in this paper.

## Data Availability

The data that support the findings of this study are available from the corresponding author upon reasonable request.
